# Characterization of macular thickness changes in Leber’s hereditary optic neuropathy by optical coherence tomography

**DOI:** 10.1186/1471-2415-14-105

**Published:** 2014-09-01

**Authors:** Yixin Zhang, Houbin Huang, Shihui Wei, Yan Gong, Hongyang Li, Yanli Dai, Shuo Zhao, Yingyun Wang, Hongxin Yan

**Affiliations:** 1 Department of Ophthalmology, Hainan Branch of Chinese PLA General Hospital, Sanya, Hainan 572013, China; 2 Department of Ophthalmology, Chinese PLA General Hospital, No.28, Fuxing Road, Beijing 100853, China

**Keywords:** Leber’s hereditary optic neuropathy, Optical coherence tomography, Retinal nerve fiber layer, Macular thickness

## Abstract

**Background:**

To characterize macular thickness (MT) changes in Leber’s hereditary optic neuropathy (LHON) patients by cirrus HD-optical coherence tomography (OCT), and to study the correlation between MT and best corrected visual acuity (BCVA).

**Methods:**

Fifty-two eyes from 52 consecutive LHON patients and 14 eyes from 14 age- and sex-matched healthy controls were scanned by OCT. Affected eyes were classified into five groups according to disease duration (1st group: ≤3 months; 2nd group: 3–6 months; 3rd group: 6–9 months; 4th group: 9–12 months; and 5th group: >12 months). MT was compared and analyzed. The correlation between BCVA and MT was calculated.

**Results:**

Less than six months after LHON onset, the cube average thickness (CAT) and the MT in the superior, nasal, inferior, and temporal quadrants of the inner ring and the MT in the nasal quadrant of the outer ring were decreased (P < 0.005); at 3–6 months onset, the MT of the temporal quadrants of the outer ring was decreased (P = 0.045); after 6 months, the MT was significantly thinner in all measurements (P < 0.01) except for the central ring. The BCVA was significantly different between each group and controls (P < 0.05), but there was no significant correlation among the five groups (P = 0.666). There was no significant correlation between the BCVA and CAT (P = 0.893).

**Conclusions:**

The MT thinned before the retinal nerve fiber layer and this occurred with a particular sequence. Our results provide potential diagnostic information for LHON.

## Background

Leber’s hereditary optic neuropathy (LHON) is a maternally inherited disease. It is clinically recognizable by the rapid, painless, bilateral loss of central vision, which usually does not manifest until young adulthood [1]. The visual acuity is usually worse than 20/400, and there is optic nerve dysfunction manifested as large and dense central or cecocentral scotomas on visual field analysis. Fundus examination in LHON may show telangiectatic capillaries and pseudoedema of the optic disc with surrounding swelling of the retinal nerve fiber layer (RNFL) [2]. More than 95% of LHON cases are caused by three point mutations of mitochondrial DNA (mtDNA): G11778A, T14484C, and G3460A. However, incomplete penetrance implies that the mtDNA mutation is a necessary but not sufficient condition for LHON, and additional genetic or environmental factors are needed to trigger the pathologic process [3]. Histopathologic descriptions of molecularly characterized LHON patients have demonstrated dramatic loss of retinal ganglion cells (RGCs) and their axons, which constitute the nerve fiber layer and optic nerve. The onset of LHON is mostly related to the papillomacular bundle (PMB) apoptosis and axonal swelling [4,5]. The small-caliber fibers of the PMB are selectively lost in a very early stage of the pathologic process. Eventually, the process extends to the rest of the nerve, leading to optic atrophy [6].

Optical coherence tomography (OCT) is a non-invasive, non-contact, *in vivo* diagnostic tool that obtains high-resolution, cross-sectional images from within the retina and enables clinicians to repetitively and reliably detect and quantify subtle changes in the RNFL and macular thickness (MT) [7,8]. OCT has been widely applied in optic nerve and retinal diseases. Current studies have shown that eyes with early LHON (E-LHON) showed a thicker RNFL, except in the temporal quadrant, and eyes with atrophic LHON (A-LHON) showed a thinner RNFL in all measurements [9]. A significant increase in RNFL thickness was detected in the temporal and inferior quadrants between the presymptomatic stage and disease onset. A significant reduction in the RNFL was detected in all but the nasal quadrants between the presymptomatic stage and the 9-month follow-up visit [10]. However, there has been no report on MT in patients with LHON. We report here MT measurements in the eyes of LHON patients using Cirrus HD-OCT. We studied the correlation between MT changes and best corrected visual acuity (BCVA).

## Methods

### Subjects

The study was approved by our hospital ethnics committee, IRB of Chinese PLA General Hospital. The ethics committee approved the relating screening, inspection, and data collection of the patients, and all subjects signed a written informed consent form. All works were undertaken following the provisions of the Declaration of Helsinki.

The patients that were diagnosed using mtDNA and confirmed as having LHON were recruited between September 2011 and April 2013 in the Department of Neuro-ophthalmology of the Chinese PLA General Hospital. All subjects underwent OCT testing and a complete ophthalmologic examination, including the following aspects: BCVA, intraocular pressure measurement, slit-lamp examination, ophthalmoscopy, visual field testing, visual evoked potential, and electroretinogram. Eyes that exhibited signs of retinal disease or other optic neuropathies were excluded. Subjects were excluded if they were unwilling to participate or if stable OCT images were not able to be obtained. Affected eyes were divided into five diagnostic groups according to disease duration (i.e., 1st group: ≤3 months; 2nd group: 3–6 months; 3rd group: 6–9 months; 4th group: 19–12 months; and 5th group: >12 months). Age- and sex-matched control subjects were recruited from the relatives of the patients and workers in our center were also recruited. They underwent OCT testing and general ophthalmologic examination. Based on the OCT results, eyes with relatively better OCT signals than others were considered as the 6th group.

### OCT procedure

**Figure 1:**
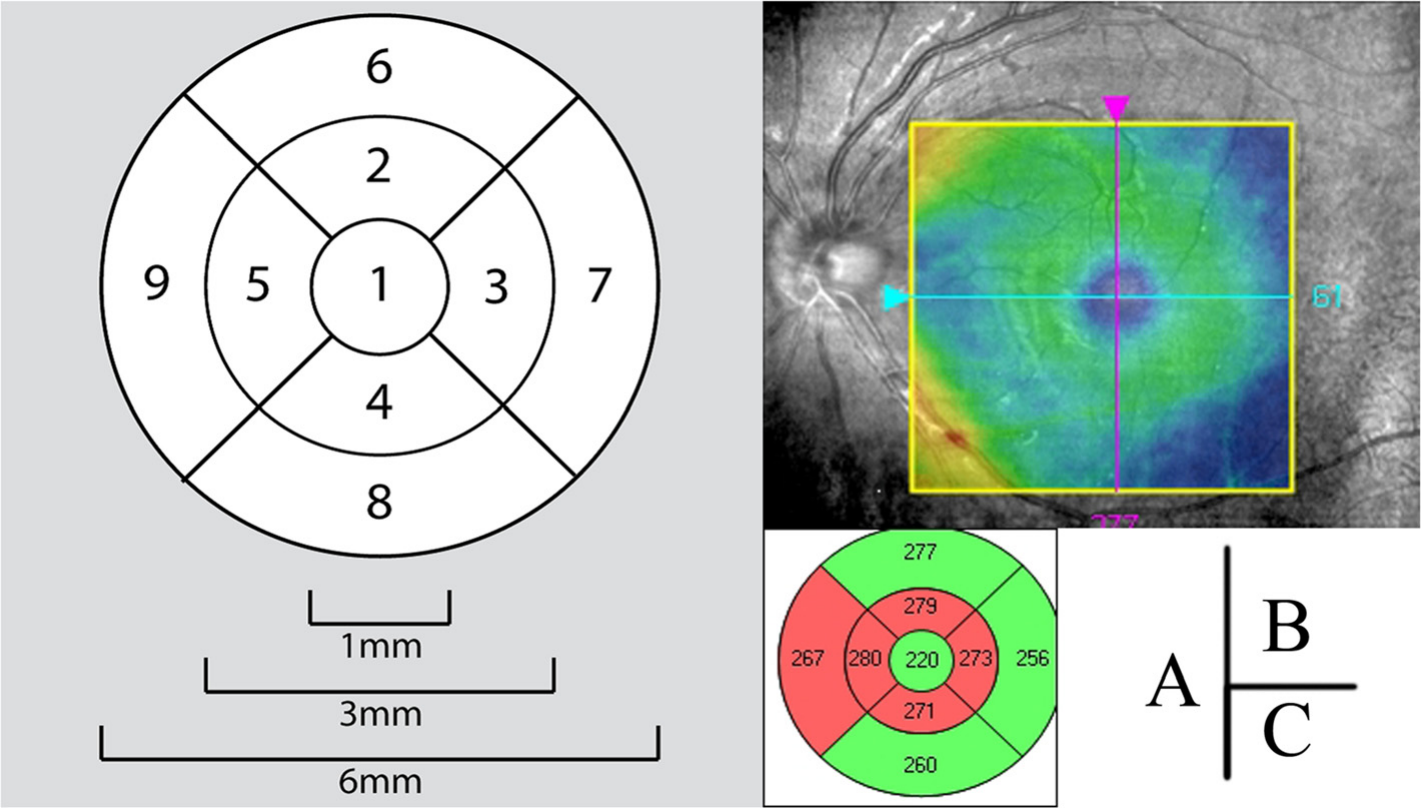
**Macula lutea thickness by optical coherence tomography. (A)** Nine macular areas describing thema ucla lutea; center ring (region 1), superior quadrant of the inner ring (region 2), inferior quadrant of the inner ring (region 4), temporal quadrant of the inner ring [region 5 (OD); region 3 (OS)], nasal quadrant of the inner ring [region 3 (OD); region 5 (OS)], superior quadrant of the outer ring (region 6), inferior quadrant of the outer ring (region 8), temporal quadrant of the outer ring [region 9 (OD); region 7 (OS)], nasal quadrant of the outer ring [region 7 (OD); region 9 (OS)]. OD = right eye; OS = left eye. **(B)** The location and range measure by OCT; **(C)** A nine macular areas measurement values by OCT in an LHON patient, the left eye macular thickness suggested that all nasal quadrants of the inner and outer rings were thinning, the center ring and superior and inferior quadrants of the outer ring were normal.

OCT scanning was performed using Cirrus HD-OCT (software version 3.0, Model 4000; Carl Zeiss Meditec, Inc., Dublin, CA, USA), which acquires real-time image scans (27,000 A-scans per second) and axial resolution (5 micron), and restructures these data as a three-dimensional cube. Information on scanning modes and image analyses were obtained from the manufacturer. MT scanning was performed using the Macular cube 200 × 200 protocol. BCVA examinations were performed using a logMAR visual testing chart.All OCT scanning was performed by an experienced operator in a darkroom. Patients with a pupil diameter less than 2 mm received mydriasis. In all cases, if a patient could see the interior fixed optotypes, he/she would be asked to look at center of the optotypes. If the subject could not fixate internally, he/she would be asked to watch the light-emitting diode in the terminal of peripheral equipment using the fellow eye. If the methods described above were infeasible, the patient was asked to move the eyes in the corresponding direction until the image of the optic disc became clear. The eyes were then rescanned and well-fixed images with OCT signals of more than 6 were saved. Macular data were evaluated by the cube average thickness (quadrant measurements of retinal thickness in a 6 mm × 6 mm volume cube between the inner limiting membrane and the retinal pigment epithelium: ILM-RPE) and the thickness map displaying measurements calculated from nine macular areas corresponding to the Early Treatment Diabetic Retinopathy Study (ETDRS; Figure 1A).

### Statistical analysis

All data were analyzed using SPSS software (Version 19.0). Variance analysis with a least significant difference, multiple comparisons, *post hoc* test was performed to compare the variations in the quantitative data of the test groups. Linear regression analysis was used to determine the correlation between the MT and BCVA. Results with a p-value less than 0.05 were considered significant.

## Results

A total of 52 eyes from 52 patients with LHON were recruited in our study, and another 14 eyes were selected successful from 14 healthy controls. Demographic data of the study cohorts are summarized in Table 1.

**Table 1:** Demographic data of patients undergoing optical coherence tomography

	**LHON patients (N = 52)**					**Normal (N = 14)**
	**≤3 months**	**3–6 months**	**6–9 months**	**9–12 months**	**>12 months**	
**Group**	**1st**	**2nd**	**3rd**	**4th**	**5th**	**6th**
Eyes	10	11	10	9	12	14
Age	18.40 ± 7.81	20.27 ± 6.31	22.90 ± 8.66	14.78 ± 5.19	23.08 ± 6.20	28.29 ± 14.64
Age of onset (years)	18.00 ± 8.17	20.09 ± 6.02	22.20 ± 8.61	14.00 ± 4.98	15.83 ± 4.59	
Male/female	10/0	11/0	9/1	8/1	11/1	12/2
BCVA	1.47 ± 0.68	1.42 ± 0.85	1.64 ± 0.66	1.31 ± 0.77	1.17 ± 0.66	0.03 ± 0.11
mtDNA mutation point (male/female)	G11778A (6/0)	G11778A (5/0)	G11778A (6/1)	G11778A (5/0)	G11778A (5/1)	
	G11778A + G3316A (1/0)	G11778A + G3644A (1/0)	G11778A + G3316A (1/0)	G11778A + T14502C (0/1)	G11778A + G3394C (1/0)	
	T14484C (1/0)	T14484C (3/0)	T14484C (1/0)	T14484C (3/0)	G11778A + G3644C (1/0)	
	G3460A (1/0)	G3460A (1/0)	G3460A (1/0)		T14484C (1/0)	
	C3497T + G3316A (1/0)	G3460A + G3316A (1/0)			G3460A (1/0)	
					T14502C (1)	
					T14502C + G3635A (1)	

The MT measured by OCT in LHON patients and controls is provided in Table 2, according to the sequence of LHON; i.e., onset and 3 months, 6 months, 9 months, and 12 months after the initial visit. Figure 1B and Figure 1C show a MT measurement result by OCT in a LHON patient.

**Table 2:** Cube average thickness in patients with different courses and mean macular thicknesses of the standard nine ETDRS subfields (μm)

		**≤3 months**	**3–6 months**	**6–9 months**	**9–12 months**	**>12 months**	**Normal**
Cube average thickness		266.90 ± 21.41	266.73 ± 15.49	252.70 ± 14.83	251.11 ± 11.10	235.92 ± 15.04	280.71 ± 8.67
Center ring	MT	226.70 ± 16.66	233.45 ± 21.17	230.10 ± 14.93	223.22 ± 22.94	225.08 ± 12.32	239.14 ± 21.39
Inner ring	MT superior	284.90 ± 24.15	287.27 ± 16.54	281.00 ± 8.29	283.00 ± 17.91	262.92 ± 16.94	325.71 ± 11.75
	MT nasal	277.80 ± 19.67	282.45 ± 13.03	276.40 ± 8.98	280.11 ± 16.97	267.83 ± 13.46	326.43 ± 15.04
	MT inferior	277.30 ± 18.48	276.48 ± 17.80	273.90 ± 11.38	277.33 ± 17.02	260.28 ± 11.67	320.00 ± 12.96
	MT temporal	275.40 ± 21.42	278.18 ± 11.14	270.10 ± 11.27	270.89 ± 13.97	253.92 ± 9.68	309.29 ± 11.51
Outer ring	MT superior	272.50 ± 21.94	270.91 ± 18.22	258.00 ± 12.68	255.22 ± 10.99	238.42 ± 8.41	279.07 ± 10.04
	MT nasal	273.00 ± 24.44	273.55 ± 19.91	260.30 ± 10.48	260.56 ± 12.04	243.50 ± 9.81	303.64 ± 11.72
	MT inferior	261.10 ± 20.55	263.64 ± 20.80	249.00 ± 17.63	241.22 ± 12.13	224.17 ± 10.56	268.14 ± 10.98
	MT temporal	255.56 ± 19.76	253.27 ± 9.27	244.13 ± 11.81	245.63 ± 9.96	230.18 ± 10.64	263.07 ± 8.58

### Comparison between healthy controls and LHON patients

#### Comparison of the MT in the center ring

The MT in the center ring had no statistically significant differences among all groups (P = 0.312).

#### Comparison of the MT in the inner and outer rings

Compared with the control group, the MT in the cube average thickness, the MT in the superior, nasal, inferior, and temporal quadrants of the inner ring and the MT in the nasal quadrant of the outer ring were thinner (P = 0.027, P = 0.000, P = 0.000, P = 0.000, P = 0.000, and P = 0.000, respectively), and no statistically significant differences were found in the MT in the superior, inferior, and temporal quadrants of the outer ring (P = 0.272, P = 0.284, and P = 0.144, respectively) within 3 months after LHON onset. Within 3–6 months, the MT in the cube average thickness, the MT in the superior, nasal, inferior, and temporal quadrants of the inner ring, and the MT in the nasal and the temporal quadrants of the outer ring were thinner (P = 0.021, P = 0.000, P = 0.000, P = 0.000, P = 0.000, P = 0.000, and P = 0.045, respectively). No significant changes were detected in the MT in the superior and inferior quadrants of the outer ring (P = 0.163 and P = 0.480, respectively). A thinner MT was detected in all measurements (P < =0.06) within 6–9 months, 9–12 months, and after 12 months after LHON initial onset.

### Correlation between the cube average thickness and BCVA

The BCVA differences among all groups showed no statistically significant differences (P = 0.666). There was no significant correlation between the BCVA and cube average thickness (P = 0.893).

## Discussion

With the development and continuous upgrading of OCT, optical coherence tomography has become one of the most efficient techniques in optic neuropathy and retinal disease research, especially in research on retina anatomy and RNFL thickness [11,12]. OCT has been widely used in ophthalmologic diseases such as glaucoma, optic neuritis, and multiple sclerosis. However, research using OCT on LHON is still limited, especially for analyzing MT in LHON.

Comparing patients with normal subjects, the MT in the center ring was not statistically significantly different in this study. In general, the central fovea of the macula has a diameter of 1.5 mm, including a thin fundus, slope of 22° and a thick edge, and there are few ganglion cells in the 0.35-mm-diameter fundus of the fovea [13]. In an OCT scan, the diameter of the center ring is around 1 mm, which allows detection of the fundus, slope, and even the part of the edge. However, the lack of ganglion cells and nerve fibers in these areas resulted in the failure of observation of ganglion cells and nerve fibers in the center ring with OCT. Moreover, apoptosis of ganglion cells and loss of axons in these areas does not cause thinning of the MT in the center ring.

**Figure 2:**
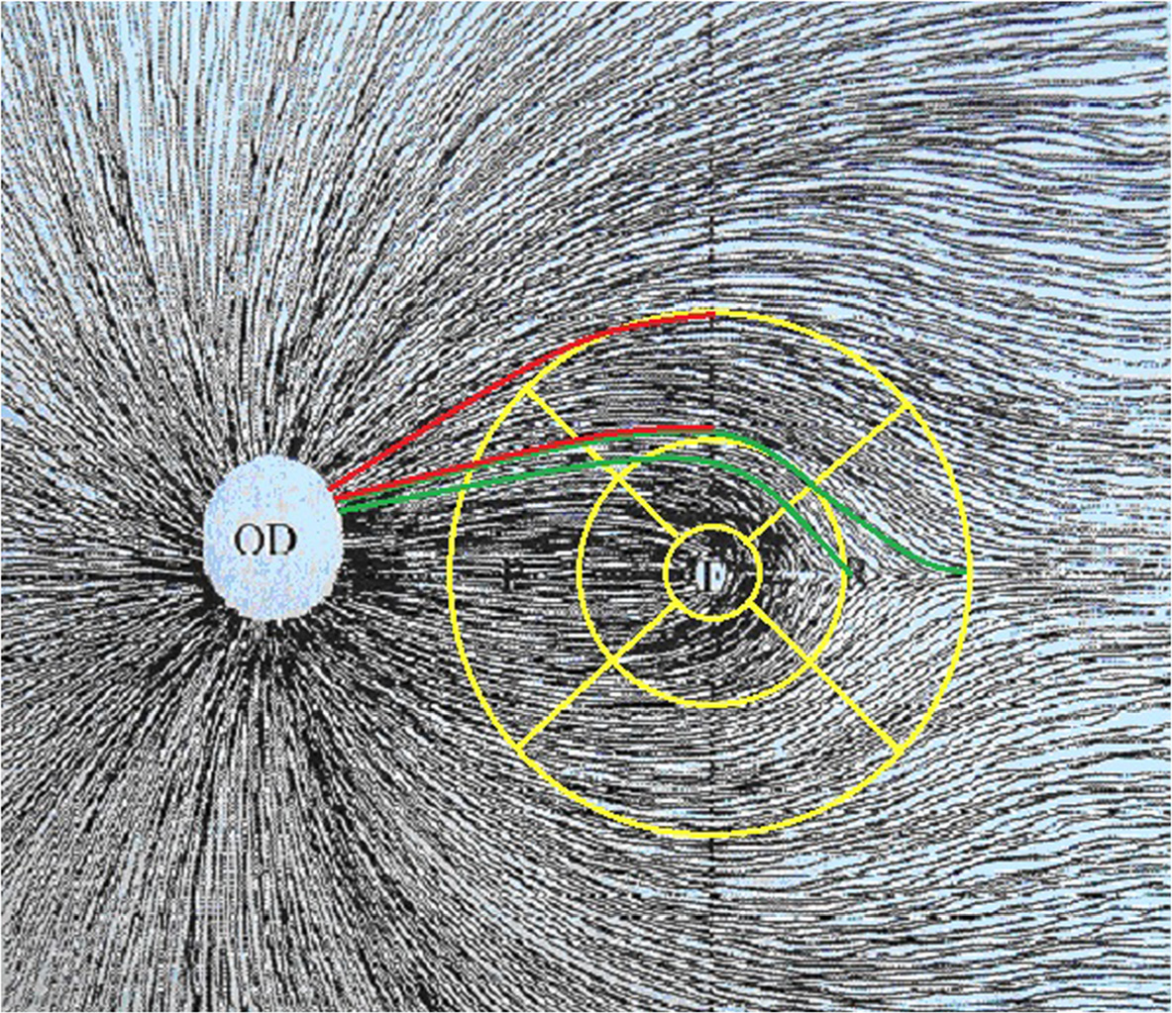
**The line feed of the retinal nerve fibers.** The line feed of nerve fibers, which originated from the ganglion cells in the superior quadrant of the outer ring is represented in the upper 2 line; while the lower two lines represents that in the temporal quadrant of the outer ring (indicating nerve fibers originating from the ganglion cells in the temporal quadrant of the outer ring closer to the papillomacular bundles).

Comparing patients with normal subjects within 3 months, the MT in the cube average thickness, in all quadrants of the inner ring and in the nasal quadrant of the outer ring were significantly thinner in this study. Previous studies suggested that the onset of LHON was mostly related to PMB apoptosis and axonal swelling [4,5] because the small-caliber fibers of the PMB were selectively lost at a very early stage of the pathologic process and the inner ring and the nasal quadrant of the outer ring were right in the area of the line feed of the PMB. Meanwhile, the nerve fibers from the RGCs of these fields projected to the temporal direction of the optic disc, which showed coherent variations of RNFL thickness in the patients with LHON. RNFL thickness in the temporal quadrant became thinner first [10]. In a previous study on the RNFL, we showed the temporal side of the RNFL exhibited a trend of thinning first, which happened in the initial 3–6 months of the disease and indicated that the thinning of the ganglion cell layer was earlier than the RNFL [14]. The perifoveal retina is the peripheral zone of the macular region. The macula has two or more layers of ganglion cells. However, when it extends to the peripheral zone, the ganglion cell layer was reduced to a single layer [15]. Therefore, the impact of retinal ganglion cell apoptosis and axonal degeneration on the MT of the inner ring is greater than that on the outer ring. The MT in the temporal quadrant of the outer ring (within 3–6 months) became thinner before the superior and inferior quadrants (within 6–9 months), which might be related to the distribution of macular ganglion cells and the line feed of the retinal nerve fibers (Figure 2). The nerve fibers from ganglion cells in the temporal quadrant of the outer ring are much closer to the PMB than those from the superior and inferior quadrants. The thinning order of the MT in different quadrants was identical to the pathogenesis mechanisms of LHON, which suggested that, in the occurrence of LHON, the pathology was observed first in the fibrils of the PMB and then extended to the outer area. Additionally, we also found that the thinning of the RNFL started in the temporal quadrant and then extended to the superior and inferior quadrants [14], which is identical with that of the MT. Except for the thinning order, the germination, growth, and projection of nerve fibers in the RNFL and the MT were also quite similar. There was no significant correlation between the BCVA and the cube average thickness. Thus, there was no direct relationship between the vision of patients with LHON and the thickness of the MT. In the early stage of LHON, the central vision of patients always deceases significantly whereas peripheral vision is unaffected, which results from the thinning of the PMB. However, most patients will gain their vision back with the development of the disease, but the cube average thickness will keep thinning at the same time, which indicates no direct relationship between vision and cube average thickness.

After the onset of LHON, macula retinae underwent atrophy and became thinner. However, the RNFL around the optic nerve became thicker first and then became thinner [10,13]. This could explain the central vision loss with a normal RNFL thickness after the onset of LHON. The cube average thickness decreased within 6 months and the MT in all quadrants became thinner within 9 months, while the mean RNFL thickness decreased after 6 months and the RNFL in the nasal quadrant showed no significant change within 9 months [9]. These results suggested that the macula thinned before the RNFL and measurements of thickness of the RNFL and MT by OCT might be a good technique for observation of disease progression and guidance of clinical treatments. Although our study has found a unique progression in MT thickness changes and provided a promising technique for clinical assessment of LHON, further study is warranted. First, the sample size in our study was relatively small, because LHON has a low incidence rate and some patients were uncooperative. Second, confounded by the insufficient sampling of our experiment, we could not conduct a thorough search for different mutation sites of the MT. Further studies should be performed to study the variability of the MT from premorbid to postmorbid in a larger sample size.

## Conclusion

Our study found that the MT thinned before the RNFL. The MT in the cube average thickness, all quadrants of the inner ring, and the nasal quadrants of the outer ring were detected as thinner first, followed by the MT in the temporal quadrant of the outer ring during 3–6 months, then the MT in the superior and inferior quadrants of the outer ring during 6–9 months. The MT in the center ring was not detected as thinner during the entire disease duration. These findings suggested that MT measurements made by OCT may be useful in the clinical assessment of LHON and differential diagnosis of LHON from other optic nerve diseases.

## Competing interests

The authors declare that they have no competing interests.

## Authors’ contribution

YXZ, HBH, and SHW defined the research theme. YG and HYL designed methods. YLD and SZ interpreted the results. YYW and HXY co-worked on associated data collection and their interpretation. All authors read and approved the final manuscript.

## Pre-publication history

The pre-publication history for this paper can be accessed here:

http://www.biomedcentral.com/1471-2415/14/105/prepub
